# Supplementary material for: Comparative multiomics analysis of cell physiological state after culture in a basket bioreactor
Source: Sci Rep. 2022 Nov 23;12:20161. doi: 10.1038/s41598-022-24687-4 (PMC9686226; doi:10.1038/s41598-022-24687-4)
Supplement: Supplementary file 1 — Supplementary Information 1. [file 41598_2022_24687_MOESM1_ESM.zip › raw data/Proteomic raw data/7.IPRenrichment/EG.vs.CG/EG.vs.CG.IPR_Enrich.scatterplot.pdf]

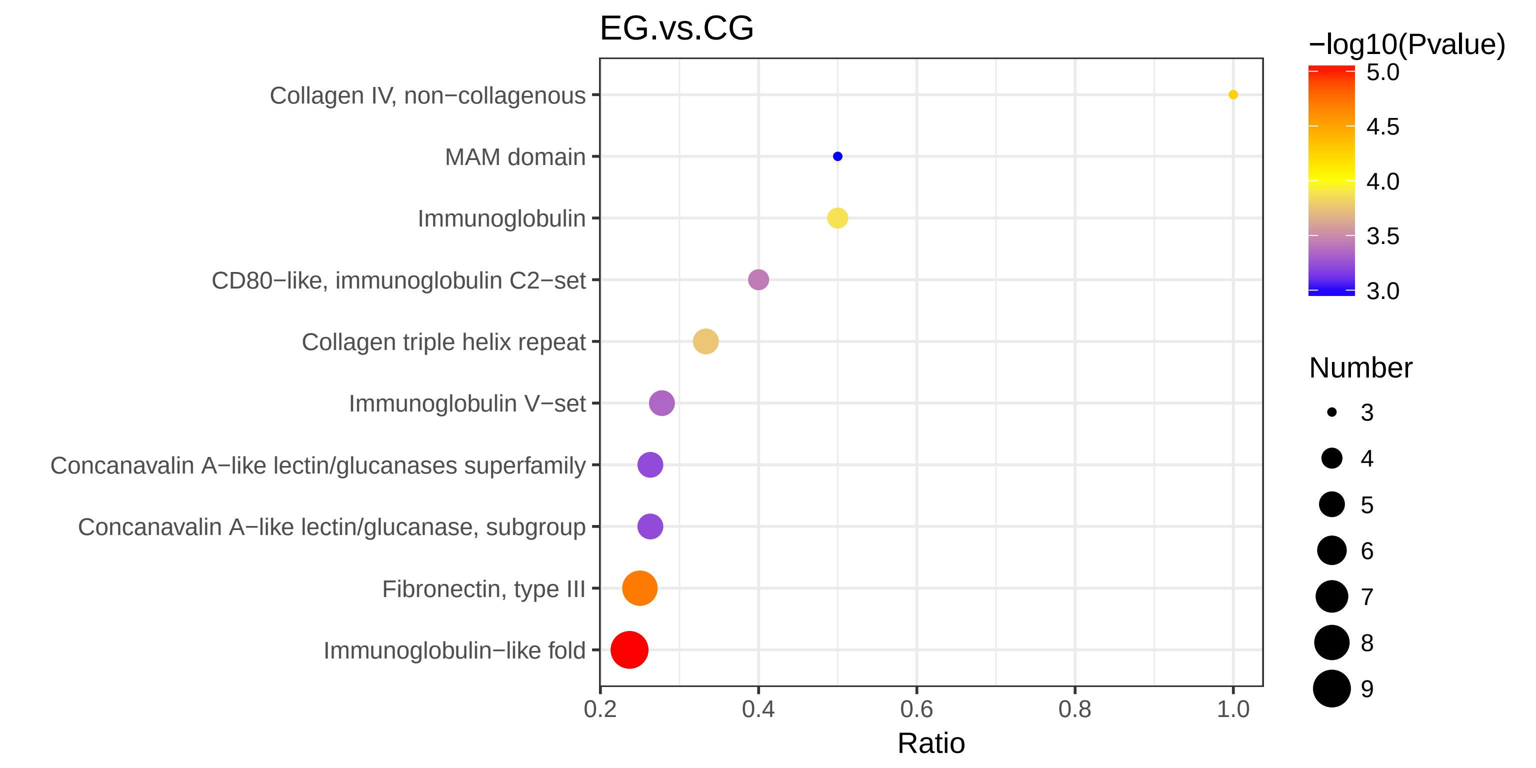
EG.vs.CG
Collagen IV, non−collagenous
MAM domain
Immunoglobulin
CD80−like, immunoglobulin C2−set
Collagen triple helix repeat
Immunoglobulin V−set
Concanavalin A−like lectin/glucanases superfamily
Concanavalin A−like lectin/glucanase, subgroup
Fibronectin, type III
Immunoglobulin−like fold
0.2
0.4
0.6
0.8
1.0
Ratio
−log10(Pvalue)
5.0
4.5
4.0
3.5
3.0
Number
3
4
5
6
7
8
9
